# Higher expression of vascular endothelial growth factor (VEGF) and its receptor VEGFR-2 (Flk-1) and metalloproteinase-9 (MMP-9) in a rat model of peritoneal endometriosis is similar to cancer diseases

**DOI:** 10.1186/1756-9966-29-4

**Published:** 2010-01-19

**Authors:** Daniel E Machado, Plínio T Berardo, Celia Y Palmero, Luiz E Nasciutti

**Affiliations:** 1 Programa de Pesquisa em Biologia Celular e do Desenvolvimento, Instituto de Ciências Biomédicas, Universidade Federal do Rio de Janeiro, Cidade Universitária-Ilha do Fundão, 21941-590 Rio de Janeiro, RJ Brazil

## Abstract

**Background:**

Endometriosis is a common disease characterized by the presence of a functional endometrium outside the uterine cavity, causing pelvic pain, dysmenorrheal, and infertility. This disease has been associated to development of different types of malignancies; therefore new blood vessels are essential for the survival of the endometrial implant. Our previous observations on humans showed that angiogenesis is predominantly found in rectosigmoid endometriosis, a deeply infiltrating disease. In this study, we have established the experimental model of rat peritoneal endometriosis to evaluate the process of angiogenesis and to compare with eutopic endometrium.

**Methods:**

We have investigated the morphological characteristics of these lesions and the vascular density, VEGF and its receptor Flk-1 and MMP-9 expression, and activated macrophage distribution, using immunohistochemistry and RT-PCR.

**Results:**

As expected, the auto-transplantation of endometrium pieces into the peritoneal cavity is a well-established method for endometriosis induction in rats. The lesions were cystic and vascularized, and demonstrated histological hallmarks of human pathology, such as endometrial glands and stroma. The vascular density and the presence of VEGF and Flk-1 and MMP-9 were significantly higher in endometriotic lesions than in eutopic endometrium, and confirmed the angiogenic potential of these lesions. We also observed an increase in the number of activated macrophages (ED-1 positive cells) in the endometriotic lesions, showing a positive correlation with VEGF.

**Conclusion:**

The present endometriosis model would be useful for investigation of the mechanisms of angiogenesis process involved in the peritoneal attachment of endometrial cells, as well as of the effects of therapeutic drugs, particularly with antiangiogenic activity.

## Background

Endometriosis is a pathology defined as the presence of endometrium-like tissue outside the uterine cavity, which consists of proliferating functional endometrial glands and stroma [1]. It is one of the most frequent gynecological diseases, and is thought to occur in 7-10% of women [2] but may even affect up to 60% of women of reproductive age with pelvic symptoms or disturbance of fertility [3]. The development and maintenance of the disease is dependent on the recruitment of blood vessels to the endometriotic lesions from pre-existing ones to guarantee oxygen and essential nutrient supply [4]. It has been shown that neovascularization is necessary for the survival of tumor implants larger than 2-3 mm^3 ^[5], and that endometriotic lesions recruit blood vessels by inducing angiogenesis [6]. In addition, epidemiological studies have shown that women with endometriosis have an increased risk of different types of malignancies, especially ovarian cancer and non-Hodgkin's lymphoma [7,8].

The development of new blood vessels is a complex dynamic process, which is characterized by a coordinated sequence of humoral and cellular interactions [9]. Upon stimulation by angiogenic growth factors, the wall of mature blood vessels becomes destabilized due to the detachment of mural cells and the degradation of the extracellular matrix that is a primordial step for the formation of new vessels. Chen et al. (2004) [10] reported higher metalloproteinase-9 (MMP-9) and lower tissue inhibitor of MMPs-1 (TIMP-1) immunostaining in ectopic and eutopic endometrium. This enables the endothelial cells to migrate into the surrounding interstitium, resulting in the formation of capillary buds and sprouts [10]. Endothelial cells behind the migrating endothelium of the sprouts proliferate so that the length and the diameter of the newly developing blood vessels increase continuously. Finally, the new vessel wall is stabilized by the attachment of mural cells, including pericytes and smooth muscle cells and the production of extracellular matrix compounds [11].

Angiogenesis is considered as a major process in the pathogenesis of endometriosis. Many factors are involved in this complex mechanism, and the vascular endothelial growth factor (VEGF) is an important mediator of angiogenesis; it is a potent endothelial cell mitogen, morphogen, and vascular permeability-inducing agent [12,13]. VEGF binds to either of two tyrosine kinase receptors, the fm5-like tyrosine kinase (flt) and the kinase domain receptor (KDR or Flk-1) [14]. Peritoneal endometriotic lesions with high proliferative activity are also accompanied by high angiogenic activity, as reflected by higher expression of VEGF-A in stroma and glandular epithelium and VEGFR-2 in blood vessels [15]. In our recent study, we showed that the vascular density and the expression of VEGF and its receptor VEGFR-2 (Flk-1) are significantly higher in deeply infiltrating endometriosis affecting the ovary, bladder and mainly the rectosigmoid, compared with the eutopic endometrium [16].

Controlled clinical analyses of angiogenesis in human endometriotic lesions are limited, because it is not possible to monitor the lesions without repeated laparoscopies. Thus, research into the fundamental mechanisms by which menstrual endometrium adheres, invades and establishes a functional vasculature to persist in an ectopic site, as well as the development of new therapeutical approaches, is best performed in experimental animal models. In contrast to humans and non-human primates, estrous animals do not shed their endometrial tissue and therefore do not develop endometriosis spontaneously. However, endometriosis can be induced by transplanting endometrial tissue to ectopic sites, and the establishment of an experimental model of endometriosis may be a good way to study the endometriosis angiogenesis process, and allow evaluation of the balance of the many factors involved [17].

In this study, we established a rat experimental model of peritoneal endometriosis, and we analyzed the vascular density and expression of VEGF and its receptor VEGFR-2 (Flk-1) and MMP-9, with the objective to evaluate the angiogenesis process and its implication in the establishment and growing of endometriosis. Our results indicated an increase of angiogenesis in endometriotic tissues similar to that observed in the human disease.

## Methods

### Animals

Animals were treated in accordance with protocols approved by the Institutional Animal Care and Use Internal Review Board of the Federal University of Rio de Janeiro (IBCCF-009/2008). Female Sprague-Dawley rats (200-250 g) with free access to water and food were included in this study, after reaching maturity at 8 weeks of age.

### Surgical Induction of Endometriosis

Twenty female rats were used in the experimental induction of endometriosis, using the method described by Vernon and Wilson (1985) [18]. Animals were anesthetized with intramuscular injection of ketamine and xylazine. The abdomen was opened through a 3-cm midline incision to expose the uterus. One uterine horn was ligated at both the uterotubal junction and the cervical end, and was removed. The segment was placed in phosphate-buffered saline at 37°C and split longitudinally, and 5 × 5-mm pieces were sectioned. These explants were then anchored onto the peritoneum on the right side of the ventral abdominal wall by nonadsorbable polypropylene sutures (Prolene 6-0; Ethicon, Piscataway, NJ). The abdomen was closed and the animals were allowed to recover from anesthesia.

The animals were divided into two groups to study the implantation and the angiogenic potential of these lesions. Group 1: 10 animals, analyzed 15 days after the surgery; and Group 2: 10 animals, analyzed 30 days after the surgery. After that, the animals were euthanized to determine the attachment and viability of endometrial explants. Also, from each experimental group, tissue samples of eutopic endometrium were obtained for establishing the control group. The surface area of the explants was measured (length × width) to the nearest 0,1 millimeter using calipers. After dissection, each sample was immediately divided into two pieces. One piece was fixed in 10% buffered formalin and embedded in paraffin for histological and immunohistochemical studies. The other piece was frozen in liquid nitrogen for RNA extraction.

### Histology and Immunohistochemistry

Formalin-fixed tissues were paraffin-embedded and cut into 4-μm-thick sections. Part of the sections were stained with Harris' hematoxylin and eosin, and examined microscopically for the presence of histological hallmarks of endometriosis, such as endometrial glands and stroma.

The other paraffin-embedded tissue sections were placed on silane-treated slides, and maintained at room temperature. After dewaxing, the sections were treated with a solution of 3% H_2_O_2 _in 0.01 mol/L phosphate-buffer saline (PBS), pH 7.5, to inhibit endogenous peroxidase activity. The slides were then immersed in 10 nmol/L citrate buffer (pH 6.0) and heated in a microwave oven for 5 minutes to retrieve masked antigens; to reduce nonspecific antibody binding; the sections were then incubated with PBS containing a 10% solution of normal goat serum and 5% bovine serum albumin for 30 minutes. Sections were incubated with the following antibodies: polyclonal antibody against von Willebrand-factor (vWF) A-082 (DakoCytomation, Carpinteria, CA) at 1:200 dilution, monoclonal antibody against α-smooth muscle actin (α-SMA) M0851 (DakoCytomation, Carpinteria, CA) at 1:100 dilution, monoclonal antibody against VEGF SC-7269 (Santa Cruz Biotechnology, Santa Cruz, CA) at 1:100 dilution, polyclonal antibody against VEGFR-2 (Flk-1) SC-6251 (Santa Cruz Biotechnology, Santa Cruz, CA) at 1:200 dilution, and monoclonal antibody against ED-1 macrophage antigen AB31630 (Abcam, Cambridge, MA) at 1:200 dilution. Incubations were carried out overnight and then revealed using LSAB2 Kit, HRP, rat (Dako-Cytomation, Carpinteria, CA) with diaminobenzidine (3,3'-diaminobenzidine tablets; Sigma, St. Louis, MO) as the chromogen and counterstained with hematoxylin. For each case, negative control slides consisted of sections incubated with antibody vehicle or no immune rabbit or mouse serum.

### Histomorphometry

All tissues were examined by two blinded observers using a 40× objective lens of a light microscope (Nikon, Tokyo, Japan) connected to a digital camera (Coolpix 990; Nikon). Ten fields of an immunostained section (von Willebrand-factor, α-SMA, VEGF, Flk-1 and ED-1) were chosen at random and captured from each specimen. Quantification was assessed on captured high-quality images (2048 × 1536 pixels buffer) using the Image Pro Plus 4.5.1 (Media Cybernetics, Silver Spring, MD). Data were stored in Adobe Photoshop, version 3.0, to enable uneven illumination and background color to be corrected. The number of cross sections of vWF and α-SMA-stained vessels and ED-1-stained macrophages was counted, and these numbers per square millimeter of the lesion were calculated, as described by Nap et al. (2004) [19].

A semiquantitative evaluation of immunohistochemical staining for VEGF and Flk-1 was performed according to the method described by Donnez et al. (1998) [20]. This method involves the analysis of the distribution and the intensity of staining within the endothelium and glandular epithelium or stroma. The histologic scores (*H*) for VEGF and Flk-1 were calculated using the formula *H *= ΣP*i*, where *i *is the intensity ranging from 0 (negative cells) to 3 (deeply staining cells) and P is the percentage of staining cells for each given *i*, with P values of 1, 2, 3, 4, and 5 indicating <15%, 15-50%, 50-85%, >85%, and 100% positive-staining cells, respectively. The staining result was expressed as mean ± standard deviations.

### Statistical Analyses

All statistical calculations were carried out using the Stat-Xact-5 software program (CYTEL Software Corporation, Cambridge, MA). The differences between groups were calculated using nonparametric analyses (Mann-Whitney *U *test). A *P *value of < 0.05 was established as statistically significant.

### Reverse transcription-polymerase chain reaction (RT-PCR)

To investigate the expression of VEGF and Flk-1 and MMP-9 in eutopic endometrium and in endometriotic lesions, RT-PCR was performed. Total RNA was extracted from the tissues in TRIzol reagent (Invitrogen, Carlsbad, CA, USA) according to the manufacturer's protocol. The purity and integrity of the RNA were checked by gel electrophoresis. One microgram of total RNA was subjected to reverse transcription with a commercially available kit (the cDNA First Chain Amplification System, GIBCO-BRL) according to the manufacturer's protocol. Amplification for VEGF cDNA was started with a 4-minute denaturation at 95°C followed by cycles of 30 seconds of denaturation at 94°C, 45 seconds of annealing at 61°C, and 45 seconds of extension at 72°C. The PCR profile for Flk-1 began with the 4-minute initial denaturation at 95°C, followed by cycles of 30 seconds of denaturation at 94°C, 45 seconds of annealing at 58°C, and 45 seconds of extension at 72°C. Amplification for MMP-9 cDNA was performed according to the following profile: initial denaturations at 94°C for 5 min, then 30 cycles at 94°C for 1 min 30 s, 63°C for 2 min and 72°C for 1 min. Transcripts were quantified after normalization with the endogenous control (GAPDH). Amplification for GAPDH cDNA was started with a 4-minute denaturation at 94°C followed by cycles of 30 seconds of denaturation at 95°C, 45 seconds of annealing at 63°C, and 45 seconds of extension at 72°C. The primers used for VEGF, Flk-1, MMP-9, and GAPDH amplification were: [1] VEGF: sense: 5'-ACC ATG AAC TTT CTG CTC-3', antisense: 5'-GGA CGG CTT GAA GAT ATA-3'; [2] Flk-1: sense: 5'-GCA CTG AAT TAT GGG AGA-3', antisense: 5'-ATG TGA TTT TCT TCT TGA TG-3'; [3] MMP-9: sense: 5'-GTT TCT GCC CCA GTG AGA ATC TC-3', antisense: 5'-TGC TGG ATG TCT TTT ATG TCG-3'; [4] GAPDH: sense: 5'-CAC CAC CAT GGA GAA GGC-3', antisense: 5'-CCA TCC ACA GTC TTC TGA-3'. Final PCR products were subjected to electrophoresis through a 2% agarose gel and stained with ethidium bromide. Semiquantitative RT-PCR was determined by agarose gel electrophoresis, GelDoc 2000 digitization, Scion Image Alpha 4.0.3.2. For each primer pair, assays were designed to detect PCR product accumulation in the middle of the linear range to facilitate their relative quantification.

## Results

### Morphological characterization of rat peritoneal endometriosis

**Figure 1:**
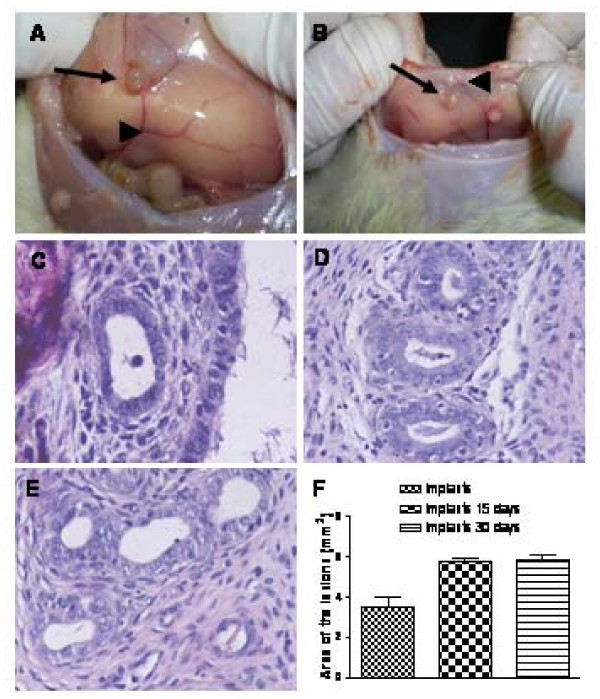
**Morphological characteristics of rat peritoneal endometriotic lesions**. Lesions after 15 days (A, C) and 30 days (B, D), eutopic endometrium (E) and histogram of implant areas (F). Most of the explants were well vascularized (arrowheads) and cystic (arrows), resembling human peritoneal endometriosis. Compared between groups, there was no detectable difference in the lesion size. Histologically, the endometriotic tissues (C, D) were similar to the eutopic endometrium (E) because they both contained endometrial glands and stromal cells, as revealed by hematoxylin and eosin coloration. Magnification × 200.

Endometriosis was induced by transplanting endometrial tissue to the rat peritoneal wall. The endometrial explants took well to the abdominal wall and produced viable implants in 18 (90%) animals of 20. The morphological characteristics of endometriotic lesions were similar in both groups (15 and 30 days after the implantation). Most of the explants were found to be well vascularized and cystic, resembling human peritoneal endometriosis (Fig. 1A, B). Compared between groups, there was no detectable difference in size; however they were larger than the tissue fragment implanted, as shown in the measurements of the macroscopic area (Fig. 1F). The histological characterization of endometriotic lesions revealed the presence of endometrial glands and stroma, very similar to that observed in eutopic endometrium (Fig. 1C, D, E). We have previously observed the endometriotic lesions 90 days after the implantation and we did not detect difference in size compared with the lesions of 30 days (data not shown).

### Microvessel density analysis

**Figure 2:**
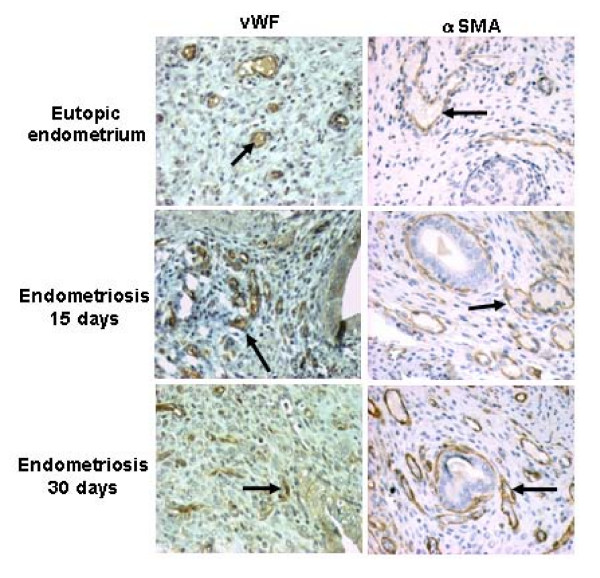
**Microvessel density was determined on the basis of vWF and αSMA-positive vessels**. The distribution of these markers was observed in the vessels located throughout the stroma, mainly around the glands. Comparing eutopic endometrium and the established endometriotic lesions, there were more positive microvessels (arrows) in the stroma around the glands in endometriosis samples after 15 and 30 days. In contrast, αSMA-positive vessels were more abundant in the lesions after 30 days. Magnification × 400.

Microvessel density was determined on the basis of vWF and αSMA-positive vessel immunodistribution. These markers were observed in the vessels located throughout the stroma, mainly around the glands. Comparison between the eutopic endometrium and the established endometriotic lesions revealed that there were more positive microvessels in the stroma around the glands in samples of endometriosis (Fig. 2). These observations were confirmed by the histomorphometry evaluation (Table 1). Although there was no significant difference between vWF in both endometriosis groups, the staining for vWF at 15 days seemed to be more intense than staining on day 30. In contrast, the immunodistribution of αSMA-positive vessels were more numerous in endometriosis samples after 30 days (Fig. 2 and Table 1).

**Table 1:** Histological scores of Von Willebrand Factor (vWF), alpha-Smooth Muscle Actin (α-SMA), Vascular Endothelial Growth Factor (VEGF), Kinase Domain Receptor (Flk-1) and ED-1-macrophage in eutopic endometrium and endometriotic lesions after15 and 30 days.

Cases	vWF (number of vessels/mm^2^)	α-SMA (number of vessels/mm^2^)	VEGF (% of positive staining cells)	Flk-1 (% of positive staining cells)	ED-1 (number of macrophage/mm^2^)
**Eutopic endometrium**	8.1 ± 0.73	5.1 ± 0.73	5.68 ± 0.10	6.46 ± 0.12	7.6 ± 1.07
**Endometriosis 15 days**	21.5 ± 1.35^a^	11.3 ± 1.15^a^	8.52 ± 0.19^a^	9.81 ± 0.11^a^	34.2 ± 0.78^a^
**Endometriosis 30 days**	20.6 ± 0.84^a^	19.2 ± 1.03^a b^	8.43 ± 0.12^a^	10.31 ± 0.18^a^	40.2 ± 1.03^a^

^a ^*P *< 0.05 (the scores for all markers are significantly higher in endometriotic lesions compared to eutopic endometrium).

^b ^*P *< 0.05 (the scores are significantly higher in endometriotic lesions after 30 days for α-SMA compared to lesions with 15 days).

Values are mean ± standard error.

### Expression of mRNA encoding for VEGF, Flk-1 and MMP-9

**Figure 3:**
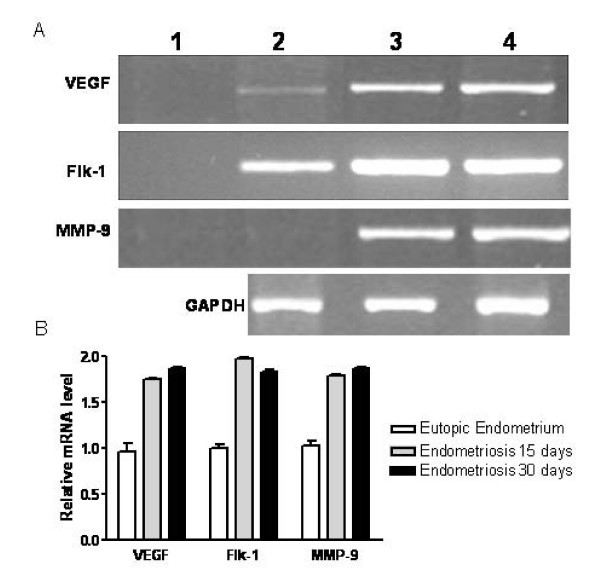
**Expression of mRNA encoding for VEGF, Flk-1 and MMP-9 in eutopic endometrium and endometriotic lesions as assayed by RT-PCR (A) and densitometry of bands (B)**. Lane 1, negative control (no cDNA); Lane 2, eutopic endometrium; Lane 3, lesions after 15 days; Lane 4, lesions after 30 days. The levels of VEGF, Flk-1 and MMP-9 mRNA transcripts in the endometriotic lesions were higher than in eutopic endometrium. Glyceraldehyde-3-phosphate dehydrogenase (GAPDH) mRNA was studied as constitutive housekeeping genes.

The mRNA transcripts of VEGF, Flk-1 and MMP-9 were analyzed in endometriotic lesions and in eutopic endometrium by quantitative RT-PCR in order to evaluate the expression of these genes. The levels of VEGF, Flk-1 and MMP-9 mRNA transcripts in the endometriotic lesions were higher than in the eutopic endometrium (Fig. 3).

### VEGF, Flk-1, and ED-1 immunodistribution

**Figure 4:**
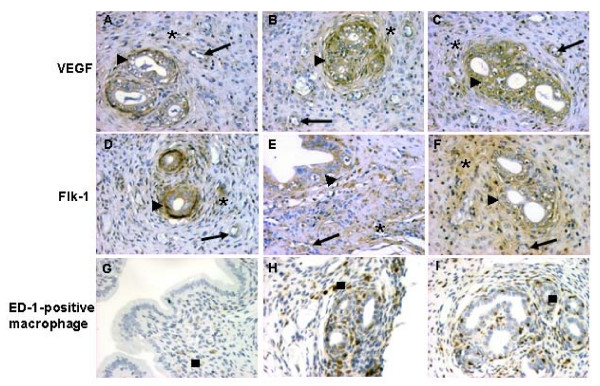
**Angiogenesis pattern of eutopic endometrium (A, D, G), and endometriotic lesions after 15 days (B, E, H) and 30 days (C, F, I)**. The immunoreactivity of VEGF and Flk-1 were detected mainly in the cytoplasm of endothelial (arrows) and glandular epithelial cells (arrowheads) but also in stromal cells (asterisks) in both eutopic and ectopic endometrial tissues. As expected, VEGF and Flk-1 immunoreactions were more abundant in endometriosis than in the eutopic endometrium. The distribution of the ED-1-positive macrophages was observed in the cells in the stroma, concentrated around the glands. There were more activated macrophages in samples of endometriosis than in eutopic endometrium (black squares). Magnification × 400.

The immunoreactivity of VEGF and Flk-1 was similar and detected focally in the cytoplasm of endothelial cells, glandular epithelial cells and diffusely in stromal cells, in both eutopic and ectopic endometrial tissues (Fig. 4). As expected, VEGF and Flk-1 immunoreactions were more intense in endometriosis than in eutopic endometrium. Comparing the endometriosis after 15 and 30 days, there were no differences in these angiogenic markers, as shown in the histological scores (Table 1).

The presence of macrophages in the tissues was analyzed using the macrophage activation marker ED-1. This immunodistribution was observed in the cells in the stroma, concentrated around the glands (Fig. 4). The numbers of activated macrophages in samples of endometriosis were higher than in eutopic endometrium. In addition, the endometriotic lesions after 30 days contained more of these cells compared to those after 15 days, as shown in Table 1.

## Discussion

The pathogenesis of endometriosis remains unclear, but it is generally considered that the development of pelvic endometriosis may be a consequence of implantation of viable endometrial tissue in ectopic sites via retrograde menstruation [21]. However, this theory fails to explain the presence of endometriosis in such remote areas as the lungs, skin, and lymph nodes. The coelomic metaplasia theory claims that formation of endometriomas in the ovary or rectovaginal endometriosis is caused by metaplasia of the coelomic epithelium, perhaps induced by environmental factors [22,23]. In addition to the retrograde flow of exfoliated endometrium, new blood vessels are essential for the survival of the implant, and therefore for the development of endometriosis. This study showed that, in a rat peritoneal endometriosis model, the angiogenic markers were related to the establishment of the lesions, confirming that this model is suitable to investigate the angiogenesis process.

The autotransplantation of uterine pieces into the peritoneal cavity is a well-established method for induction of endometriosis in rats [18,24]. In the present study, this model of autologous endometrial explants was established at 15 days in 18 (90%) animals of 20, and the explants developed into large, ovoid, fluid-filled, well vascularized, cystic structures composed of endometrial elements. Any difference was observed in the macroscopic aspect of these cystic structures on 30 days, and also after that (90 days, data not shown). In addition, we have shown that these ectopic endometrium fragments also showed histological characteristics of the human disease, including highly vascularized lesions containing endometrial glands and stroma. These results were in agreement with those of Dogan et al. (2004) [25], who reported that the endometrial explants produced viable implants in 26 of 30 animals (86.6%), and that most of the explants were well vascularized.

Analyses of the assessed microvessel density demonstrated that angiogenesis is higher in endometriotic lesions compared with the eutopic endometrium. Microvessel density was determined on the basis of vWF and α-SMA-positive vessels. The distribution of these vessel markers was more positive in stroma around the glands in samples of endometriosis. Although no significant difference was observed between the vWF positive vessels in the two groups, the immunoreaction seemed to be more intense on day 15. It could be related to the microvessel size and that the endothelial cell might not be adjacent to other pericyte or vice versa. By other hand, the α-SMA-positive vessels were more numerous in samples of endometriosis at day 30 than at day 15. This difference is related to the fact that the most of the blood vessels are mature, as illustrated by their association with αSMA-positive pericytes [4]. These observations indicated that the development of new vessels is necessary for the establishment and the maintenance of the endometriotic lesions, and also that the neovessels formed were more mature in endometriosis after 30 days. Using the same markers in the nude-mouse model of endometriosis, Nap et al. (2004) [19] demonstrated that the development of new blood vessels remains of pivotal importance for the maintenance and growth of endometriosis.

One of the main characteristics of endometriosis is its inflammatory nature. It has been shown that cytokines released from immune cells play an important role in the pathogenesis of endometriosis, and many of these cytokines possess angiogenic activity [26,27]. VEGF is the most-prominent and most-studied proangiogenic factor in endometriosis, and it is widely believed that VEGF is the main stimulus for angiogenesis and increased vessel permeability in this disease [6]. Its activity depends on its binding to different receptors, such as VEGFR-2 (Flk-1). In our model, we were able to demonstrate that the expression of VEGF and Flk-1 is enhanced in endometriotic lesions as compared with controls. Their immunodistributions were observed focally in the cytoplasm of endothelial and glandular epithelial cells and diffusely in stromal cells, and were more intense in ectopic endometrial tissues. It was also observed that the number of activated macrophages (ED-1 positive cells) increased in endometriotic lesions. These results are in agreement with other studies that have shown that VEGF is strongly expressed by endometriotic lesions and activated macrophages [12,28]. Significantly increased VEGF levels have been found in the peritoneal fluid and lesions of endometriosis patients compared to controls or eutopic endometrium, respectively [20]. These authors discovered that red, highly active endometriotic lesions contain the highest VEGF concentrations. In addition, Wang et al. (2005) [29] reported a higher Flk-1 expression in endometriosis lesions of the peritoneal and abdominal wall, which may have been associated with neovascularization.

Peritoneal macrophages and activated lymphocytes seem to play an integral role in the secretion of proinflammatory/proangiogenic cytokines. For example, in patients with endometriosis, interleukin-1β (IL-1β) is produced by activated macrophages and results in the increased expression of VEGF [24]. In a mouse model of endometriosis, it was reported that interleukin-6 (IL-6) together with tumor necrosis factor alpha (TNF-α) was secreted by macrophages, and resulted in upregulation of VEGF from infiltrating neutrophils and macrophages [30]. These data and our results support the idea that the microenvironment of endometriosis is a locale of important secretion of angiogenic factors that play a key role in the establishment and maintenance of endometriotic lesions, and suggest that the balance of these local pro-antiangiogenic factors and cytokines may determine whether endometriotic lesions develop and grow. In this context, the behavior of endometriosis tissue is very similar to that observed in tumor growth [31].

Several studies have indicated endometriosis as a risk factor and various histological and molecular genetic studies have even indicated that endometriosis may transform into cancer or that it could be considered a precursor of cancer [32-34]. Goumenou et al. [35], by microsatellite analysis, demonstrated that loss of heterozygosity on p16(Ink4), GALT, and p53, as well as on APOA2, a region frequently lost in ovarian cancer, occurs in endometriosis, even in stage II of the disease. The occurrence of such genomic alterations may represent, therefore, important events in the development of endometriosis. However, despite the histological and epidemiological evidence linking endometriosis and ovarian cancer, it is still not clear if endometriosis is a real precursor of ovarian cancer, or whether there is an indirect link involving common environmental, immunological, hormonal or genetic factors [35]. It has been clearly demonstrated that activation of a mutated K-ras gene is a fundamental step in the genesis and progression of ovarian cancer [36]. Further genetic studies are required for delineation of the risk of several malignancies and in particular of ovarian cancer in women with endometriosis.

The invasive properties of endometrium are also related to the increase of its proteolytic activity, resulting in the development of endometriosis. Chung et al. (2001) [37] showed that ectopic endometrium expressed significantly higher levels of MMP-9 mRNA and lower levels of TIMP-3 mRNA, compared to eutopic endometrium from normal and endometriosis patients. By immunohistochemistry, greater expression of MMP-9 and less expression of TIMP-1 in ectopic endometrium than in eutopic endometrium was also observed [10]. Recently, it was demonstrated in mice that the treatment of 15-Epi-lipoxin A_4 _(LXA_4_) may inhibit the progression of endometriosis possibly by lowering the concentrations and the activities of MMP-2 and MMP-9 [38]. In our model, MMP-9 mRNA expression, as expected, was greater in endometriotic lesions than in eutopic endometrium. Our results indicate a direct role for MMPs in the ability of rat endometrium to establish ectopic lesions within the peritoneum. By other hand, it is known that proteoglycans play an important role in the maintenance of vascular integrity. Kirn-Safran et al. (2008) [39] showed that proteoglycans are involved in angiogenesis by presenting and modulating a wide range of growth factors such as fibroblast growth factor-2 and -10 and VEGF on their glycosaminoglycan (GAG) side-chains. Recently, we have demonstrated that chondroitin sulfate (CS) GAG was the dominant sulfated GAG present in stroma of deeply infiltrating endometriosis lesion foci [40], as also observed in eutopic endometrium [41]. Taken together, these studies suggest that the high concentration of CS in endometriosis could be related to the angiogenesis process, and reinforce the importance of extracellular matrix metalloproteinases in the progression of endometriosis.

Animal models of endometriosis are of extreme value and indispensable for the evaluation of pathophysiological mechanisms underlying the development of this prevalent gynaecological disease. Other possible and important use for this method is to test the angiogenic therapy for endometriosis. Although there are disadvantages in extrapolating data across species, it is still possible to utilize animal models to study events involved in the pathogenesis of endometriosis that are not accessible in humans. Rat endometriotic tissues and cells perform similarly to human endometriotic cells, as revealed in this study. While the rat model for endometriosis has been used to identify effects of ectopic endometrial tissue adhesion and growth, the mechanisms eliciting these effects remain elusive. In general, animal models will help to develop novel non-invasive diagnostic tools and improved therapeutical approaches for improved treatment of endometriosis in women.

## Conclusions

Here we originally showed that the pattern of angiogenic process in rat endometriosis is very similar to human disease. Despite recent advances in the field, there is still only a limited amount of knowledge about the mechanisms regulating the complex dynamic process of blood-vessel development in endometriotic lesions. The introduction of sophisticated *in vivo *models of peritoneal and extra-peritoneal endometriosis, which allow for detailed monitoring of angiogenesis within endometriotic lesions under standardized conditions, certainly will help to clarify these mechanisms. Finally, it certainly would be important to better understand the angiogenesis process in a tumor growth.

## Competing interests

The authors declare that they have no competing interests.

## Authors' contributions

DEM participated in the design, data acquisition, manuscript writing, carried out statistical analyses and have given final approval of the version to be published. PTB participated in study design and revised manuscript. CYP performed data analysis and helped to draft the manuscript. LEN supervised the design of the experiments and analyzed and interpreted of data. All authors approved the final manuscript.
